# Preventing the Progression of Myopia in Children—A Review of the Past Decade

**DOI:** 10.3390/medicina59101859

**Published:** 2023-10-19

**Authors:** Emilia Wnękowicz-Augustyn, Sławomir Teper, Edward Wylęgała

**Affiliations:** 1 Chair and Clinical Department of Ophthalmology, Faculty of Medical Sciences in Zabrze, Medical University of Silesia, Okręgowy Szpital Kolejowy, Panewnicka 65, 40-760 Katowice, Poland; slawomir.teper@sum.edu.pl (S.T.); ewylegala@sum.edu.pl (E.W.); 2 Municipal Hospital Group, Truchana 7, 41-500 Chorzów, Poland; 3 Eye and Optics Center Augmed, Łabędzka 20d, 44-100 Gliwice, Poland

**Keywords:** myopia control, myopia prevention, defocus-incorporated multiple-segment spectacle lenses (DIMSsl), repeated low-level red-light (RLRL) therapy, combined low-dose atropine and orthokeratology lenses

## Abstract

The growing incidence of myopia worldwide justifies the search for efficient methods of myopia prevention. Numerous pharmacological, optical, and lifestyle measures have already been utilized, but there remains a need to explore more practical and predictable methods for myopia control. This paper presents a review of the most recent studies on the prevention of myopia progression using defocus-incorporated multiple-segment spectacle lenses (DIMSsl), repeated low-level red-light (RLRL) therapy, and a combination of low-dose atropine (0.01%) with orthokeratology lenses.

## 1. Introduction

Myopia control is a topic of widespread discussion among ophthalmologists, optometrists, and other eye care specialists. There is a growing incidence of myopia observed worldwide. Considering geographical prevalence, myopia is particularly prevalent in industrialized East Asian regions [1]. 

A meta-analysis conducted by Holden et al. estimated that, by the year 2050, nearly 50% of the global population will have myopia, with approximately 10% developing high myopia [2]. Myopia can lead to complications such as staphyloma, myopic maculopathy, myopic choroidal neovascularization (CNV), peripheral retinal degenerative changes, rhegmatogenous retinal detachment, optic disc changes, and glaucoma [3]. Thus, preventing the progression of myopia also helps prevent these associated comorbidities. 

Numerous medical and environmental risk factors for myopia have been identified. The association between myopia prevalence and parental myopia is well documented [4]. Additionally, several twin-eye studies support the importance of genetic factors in myopia occurrence [5]. Researchers have discussed a multitude of other potential risk factors for myopia, for example, the level of education, time spent near work, accommodation and convergence function, and time spent outdoors. An interesting question is how does the time spent outdoors protect against myopia? Lingham G. et al. reviewed the evidence for and against eight features of spending time outdoors: brighter light, reduced peripheral defocus, higher vitamin D levels, differing chromatic spectrum of light, higher physical activity, entrained circadian rhythms, less time spent on near work, and greater high spatial frequency (SF) energies [6].

In the pathomechanism of myopia, we understand that not only the axial length of the eye but also the structure of the anterior segment of the eye plays a significant role. Nevertheless, it is the axial length that is a key parameter for the progression of myopia [7]. A large number of laboratory and clinical studies support the theory of the biochemical transformation of retinal image defocus, resulting in axial elongation of the eye [8]. As our knowledge of the risk factors as well as pathophysiological and biological mechanisms of myopia advances, new insights are emerging for the prevention of myopia progression.

Studies in the literature describe various methods for myopia control. A network meta-analysis by Huang et al. [9] lists several interventions for myopia control in children, including high-dose atropine (1% or 0.5%), low-dose atropine (0.01%), moderate-dose atropine (0.1%), bifocal spectacle lenses, cyclopentolate, increased outdoor activities, orthokeratology, progressive addition spectacle lenses, prismatic bifocal spectacle lenses, peripheral defocus modifying contact lenses, peripheral defocus-modifying spectacle lenses, pirenzepine, rigid gas-permeable contact lenses, soft contact lenses, single-vision spectacle lenses, timolol, and under-corrected single-vision spectacle lenses. The aforementioned network meta-analysis included randomized controlled trials with a minimum duration of one year, spanning from inception to August 2014. More recent research describes additional interventions for myopia control, such as defocus-incorporated multiple-segment spectacle lenses [10,11,12,13,14,15,16], repeated low-level red-light therapy [17,18,19,20,21,22,23], and a combination of orthokeratology and low-dose atropine (0.01%) [24,25,26,27,28,29,30,31,32].

Defocus-incorporated multiple-segment spectacle lenses (DIMSsl) are designed to correct refractive errors while simultaneously preventing the progression of myopia. Each DIMSsl consists of a central zone for distance refractive correction and a surrounding zone comprising approximately 400 multiple defocus segments that create myopic defocus. Animal studies have demonstrated that imposed myopic defocus inhibits eye elongation, whereas hyperopic defocus promotes eye elongation [33].

Repeated low-level red-light therapy involves using a device that emits low-level red light with a wavelength of 650+/−10 nm. The intervention requires looking into the device twice a day for 3 min, with a minimum 4-h interval between sessions. Parental supervision is necessary during the therapy [21].

Orthokeratology and atropine eyedrops have separately demonstrated efficacy in slowing the progression of myopia [9]. The mechanisms of action for these interventions are different and not yet fully understood.

## 2. Materials and Methods

**Figure 1:**
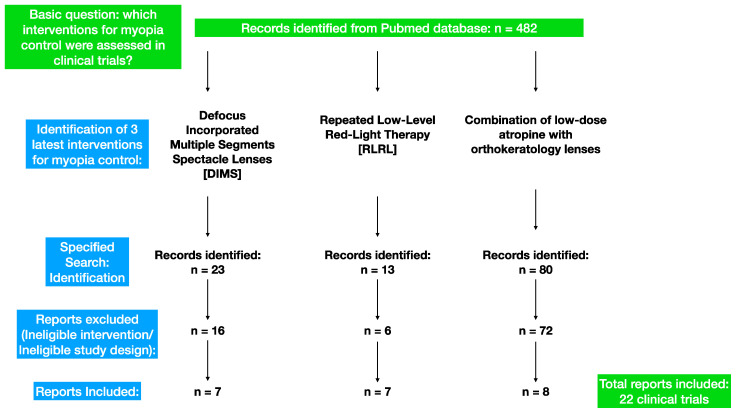
Search strategy.

The current paper provides a review of the pertinent research on the prevention of myopia progression in children using three interventions: defocus-incorporated multiple-segment spectacle lenses (DIMSsl), repeated low-level red-light (RLRL) therapy, and a combination of low-dose atropine (0.01%) with orthokeratology lenses. We utilized the PubMed database, and only peer-reviewed clinical trials published in English between the years 2011 and 2023 were considered for inclusion (Table 1, Figure 1).

## 3. The Efficacy and Safety of Defocus-Incorporated Multiple-Segment Spectacle Lenses in the Prevention of Myopia Progression

The first double-masked clinical trial assessing the efficacy of defocus-incorporated spectacle lenses (DIMSsl) was published in 2020 [12]. The study included 183 Chinese children with myopia ranging from −1.0 to −5.0 diopters and astigmatism below 1.5 diopters, aged between 8 and 13 years. Following randomization, 93 children received DIMSsl, while 90 children received single-vision spectacle lenses (SVsl). The trial lasted for 2 years, and assessments were conducted at 6-month intervals, including cycloplegic refraction and axial length of the eye. Of the initial participants, 160 completed the study. Over the course of two years, the changes in cycloplegic refraction for both groups were as follows: 0.41 ± 0.06 D in the DIMSsl group and 0.85 ± 0.08 D in the SVsl group. The mean axial elongation was 0.21 ± 0.02 mm in the DIMSsl group and 0.55 ± 0.02 mm in the SVsl group. In comparison, the myopia progression in the DIMSsl group was 52% slower than in the SVsl group, and the DIMSsl group had 62% less axial elongation than the SVsl group. Myopia control with spectacle lenses is considered a noninvasive approach. No adverse events related to the intervention were reported in this study. The authors also assessed the visual performance of DIMSsl and SVsl users in terms of parameters such as visual acuity (near and distant), the amplitude of accommodation (monocular and binocular), lag of accommodation, and stereopsis. Only the difference in stereopsis was statistically significant, but the clinical significance was negligible (5 s of arc).

In another publication from 2020, [16] documented the changes in relative peripheral refraction associated with myopia progression in the same group of patients. Central refraction and peripheral refraction at six retinal points (10°, 20°, and 30° nasally and temporally) were measured every 6 months, along with axial length measurements after cycloplegia. In the SVsl group, asymmetry between nasal and temporal retina myopic shifts was observed, while the DIMSsl group exhibited a constant and symmetrical relative peripheral refraction profile.

The visual function of the same group of patients was described in detail in a paper published in 2020 by Lam et al. [11]. The study included 160 participants who completed a 2-year trial, with 79 wearing DIMSsl and 81 wearing SVsl. Visual function was assessed by measuring distance and near best-corrected visual acuity measured monocularly, distance and near phoria, the monocular and binocular amplitude of accommodation, lag of accommodation, and stereopsis at baseline and every 6-month interval over 2 years. After two years, both groups showed a slight improvement in high contrast visual acuity: −0.09 ± 0.07 logMAR for DIMSsl wearers and −0.07 ± 0.06 logMAR for SVsl wearers. Accommodative lag was significantly reduced in both groups, and stereo-acuity improved. Distance and near phoria showed no significant changes from baseline in either group.

In 2022, Lam et al. published the results of a 3-year follow-up study [10]. Their study included 128 children who had participated in the previous 2-year trial. Those who wore DIMSsl in the previous trial continued with DIMSsl, while the SVsl group switched to DIMSsl. Refraction after cycloplegia and axial length were assessed at 6-month intervals. Both groups were compared to a new historical control group, obtained by reviewing the clinical records of the optometry clinic. Over 3 years, the mean changes in spherical equivalent refraction (SER) were −0.52 ± 0.69 D for the DIMS group and −0.92 ± 0.81 D for the SVsl group switched to DIMSsl. The mean changes in axial length over 3 years were 0.31 ± 0.26 mm for the DIMSsl group and 0.57 ± 0.33 mm for the SVsl group switched to DIMSsl.

In 2023, Zhang et al. published the results of the continued observation of changes in relative peripheral refraction associated with myopia progression [16]. The study included 128 children who continued to wear DIMScl (n = 65) for 1 year after the previous 2-year trial and those who switched to DIMSsl after 2 years of using SVsl (n = 55). The authors observed a constant and symmetrical peripheral refraction profile in the DIMSsl group. Within the SVsl group in the first 2 years, significant increases in hyperopic relative peripheral refraction (RPR) were noted at 20° nasal. After switching to DIMSsl in the third year, there were significant reductions in hyperopic RPR at 20° nasal (mean difference: −1.14 ± 1.93 D, *p* < 0.0001) and 30° nasal (mean difference: −1.07 ± 1.17 D, *p* < 0.0001).

A clinical trial conducted by Lam et al. and published in 2023 followed 90 Chinese children for a period of 6 years [13]. The mean age of the participants at enrollment was approximately 10 years old. The study involved four different groups:

- Group 1 (36 children) wore defocus-incorporated multiple-segment spectacle lenses (DIMSsl) for the entire 6-year duration;
- Group 2 (14 children) wore DIMSsl for the first 3.5 years and then switched to single-vision lenses (SVsl);
- Group 3 (22 children) wore SVsl for the first 2 years and then switched to DIMSsl for the remaining 4 years;
- Group 4 (18 children) wore SVsl for the first 2 years, then DIMSsl for 1.5 years, and finally switched back to SVsl for the last 2.5 years.

The main measured outcomes were changes in axial length (AXL) and cycloplegic refraction. The spherical equivalent refraction (SER) at baseline and the 6-year follow-up for each group were as follows:

- Group 1: SER −3.04 ± 0.89 D/−3.69 ± 1.42 D;
- Group 2: SER −2.98 ± 1.13 D/−4.28 ± 1.15 D;
- Group 3: SER −2.68 ± 0.88 D/−3.92 ± 1.18 D;
- Group 4: SER −2.65 ± 1.18 D/−3.87 ± 1.53 D.

The AXL measurements at baseline and the 6-year follow-up for each group were as follows:

- Group 1: AXL 24.68 ± 0.76 mm/25.28 ± 0.81 mm;
- Group 2: AXL 25.00 ± 0.80 mm/25.71 ± 0.69 mm;
- Group 3: AXL 24.62 ± 0.79 mm/25.43 ± 1.01 mm;
- Group 4: AXL 24.42 ± 0.86 mm/25.14 ± 0.87 mm.

These results indicate that there was no rebound effect after stopping the use of DIMSsl.

A retrospective study conducted by [14], which aimed to assess the effectiveness of DIMSsl in clinical settings, involved children aged 6 to 16 years old. After propensity score matching, data from 2240 pairs (1-year observation) and 735 pairs (2-year observation) were analyzed. The results confirmed the effectiveness of DIMSsl in slowing the progression of myopia compared to SVsl in clinical settings. The spherical equivalent progression in the first year was −0.50 ± 0.43 D for DIMSsl and −0.77 ± 0.58 D for SVsl (*p* < 0.001). In the second year, the spherical equivalent progression was −0.88 ± 0.62 D for DIMSsl and −1.23 ± 0.76 D for SVsl (*p* < 0.001). These findings provide further evidence of the effectiveness of DIMSsl in slowing myopia progression. 

**Figure 2:**
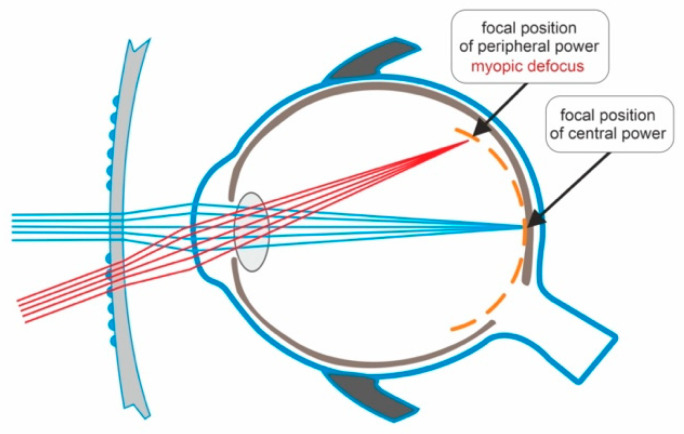
Light passing through the central zone of the DIMSsl creates a clear image on the retina. Light passing through the peripheral part of the DIMSsl creates myopic defocus on the peripheral retina.

Figure 2 depicts the mechanism of action of defocus-incorporated multiple-segments spectacle lens. 

Table 2 summarises the primary information from studies on the efficacy and safety of defocus-incorporated multiple-segments specatacle lenses. 

## 4. The Efficacy and Safety of Low-Intensity Red-Light Therapy in the Prevention of Myopia Progression

In 2022, Jian Y. et al. published a multicenter, randomized, parallel-group, single-blind clinical trial [21]. The study included 264 children aged 8–13 years with myopia ranging from −1.0 to −5.0 D cycloplegic refraction. After randomization, the sample size was as follows: 117 children in the repeated low-level red-light (RLRL) therapy group and 129 children in the single-vision spectacle lenses (SVsl) group. Cycloplegic refraction and axial length were measured at baseline and at 1-, 3-, 6-, and 12-month follow-up visits. In the RLRL group, the intervention involved repeated low-level red-light therapy using a desktop light therapy device at home, with parental supervision required. Each session lasted 3 min and was repeated twice a day, five days per week. The main results of the study were as follows: The adjusted 12-month axial elongation for the RLRL group was 0.13 mm (95% CI, 0.09–0.17 mm), and for the SVsl group, it was 0.38 mm (95% CI, 0.34–0.42 mm). The adjusted 12-month spherical equivalent refraction (SER) for the RLRL group was −0.20 D (95% CI, −0.29 to −0.11 D), and for the SVsl group, it was −0.79 D (95% CI, −0.88 to −0.69 D).

In 2023, Dong J. et al. conducted a double-masked clinical trial on repeated low-level red-light (RLRL) therapy involving 112 Chinese myopic children aged 7 to 12 years [19]. The children were divided into two groups: The RLRL group with 56 children and the sham device control group with 55 children. In the control group, a sham device with 10% of the power of the original device was used. Each session of RLRL therapy lasted 3 min and was repeated twice a day for a duration of 6 months. The mean change in spherical equivalent refraction (SER) over 6 months was as follows: −0.06 ± 0.03 D for the RLRL group and −0.11 ± 0.33 D for the sham device control group. The mean change in axial length (AL) over 6 months was −0.02 ± 0.11 D for the RLRL group and −0.13 ± 0.10 D for the sham device control group. No treatment-related adverse events were reported during the study.

In 2022, Xiong R. et al. published a prospective, post-trial follow-up study or real-world study (RWS) [22]. After completing a 1-year randomized controlled trial (RCT), the participants were invited to voluntarily participate in the real-world study. A total of 114 participants were enrolled and divided into four groups: SVS-SVS group (*n* = 41), SVS-RLRL group (*n* = 10), RLRL-SVS group (*n* = 52), and RLRL-RLRL group (*n* = 11). Cycloplegic refraction and axial length were measured at the 24-month mark from the beginning of the RCT. Over the 2-year period, the mean change in axial length (AXL) and spherical equivalent refraction (SER) were found to be the smallest in the RLRL-RLRL group. However, a modest rebound effect was observed after the cessation of treatment.

In a secondary analysis of data from a multicenter randomized controlled trial (RCT), Xiong R. et al. investigated the measurements of macular choroidal thickness (mCT) using swept-source optical coherence tomography (SS-OCT) and its associations with myopia control [23]. The study also assessed other variables at 1, 3, 6, and 12 months, including visual acuity, axial length, spherical equivalent refraction (SER), and treatment compliance. The authors aimed to determine the predictive value of different covariates for myopia control. They constructed models that included only changes in mCT at 3 months and evaluated their ability to predict good myopia control over a 12-month period. These models demonstrated acceptable predictive discrimination for myopia control.

In 2022, Chen Y. et al. conducted an RCT comparing the efficacy of repeated low-level red-light (RLRL) therapy with low-dose atropine for myopia control [18]. The study included 62 children aged 7 to 15 years who were randomly assigned to receive RLRL therapy or atropine 0.01%. Each group consisted of 31 participants. Axial length and cycloplegic spherical equivalent refraction were monitored at 1, 3, 6, and 12 months. The results indicated that RLRL therapy was effective in controlling myopia progression over one year compared to low-dose atropine eye drops.

Another RCT published in 2023 by Chen H. et al. analyzed the efficacy of low-intensity red light (LRL) therapy compared to single-focus spectacles (SFSs) for myopia control [17]. The study included 51 children in the LRL group and 51 children in the SFS group, aged 6 to 13 years, with myopia ranging from −0.75 to −6.0 diopters of cycloplegic spherical equivalent refraction. The treatment phase lasted for 12 months, followed by a 3-month washout phase. LRL therapy was administered twice a day, with each session lasting 3 min. Ophthalmic examinations, including assessments of axial length (AL), spherical equivalent refraction (SER), subfoveal choroidal thickness (SFCT), and accommodative function, were conducted at 3, 6, 9, 12, and 15 months. At the end of the 12-month trial, 46 children in the LRL group and 40 children in the SFS group completed the study. The AXL elongation at 12 months for the LRL group was 0.01 mm (95% CI 0.05–0.07 mm), while for the SFS group, it was 0.39 mm (95% CI 0.33–0.45 mm). The SER progression at 12 months for the LRL group was 0.05 D (95% CI 0.08–0.19 D), whereas for the SFS group, it was 0.64 D (95% CI 0.78–0.51 D). Changes in SFCT showed thickening in the first 3 months for the LRL group, followed by relative stability in the subsequent months, while the SFS group exhibited progressive thinning of SFCT. Accommodative function was assessed through measurements of the amplitude of accommodation (AA), accommodative response (AR), accommodative facility (AF), positive relative accommodation (PRA), and negative relative accommodation (NRA). The LRL group demonstrated more negative accommodative response and positive relative accommodation than the SFS group. 

He X. et al. conducted a study to assess the effectiveness of repeated low-level red-light (RLRL) therapy in children with premyopia, defined as a cycloplegic spherical equivalent refraction of −0.5 D to 0.5 D in the more myopic eye. The inclusion criteria also required at least one parent to have a spherical equivalent refraction of −3.0 diopters or less in either eye [20]. The study enrolled pupils in grades 1–4 from 10 primary schools in Shanghai. Participants assigned to the treatment group received two RLRL therapy sessions lasting 3 min each, daily for 5 days per week. The sessions were conducted at school, except during winter and summer vacations when they took place at home. The main outcome measure was the incidence of myopia after 12 months, which was 40.8% (49 out of 120) in the RLRL group and 61.3% (68 out of 111) in the control group. Additional results showed that RLRL intervention significantly reduced spherical equivalent refraction (SER) and axial length (myopic shifts). No adverse effects on visual acuity or structural damage were observed on optical coherence tomography (OCT) in the intervention group.

**Figure 3:**
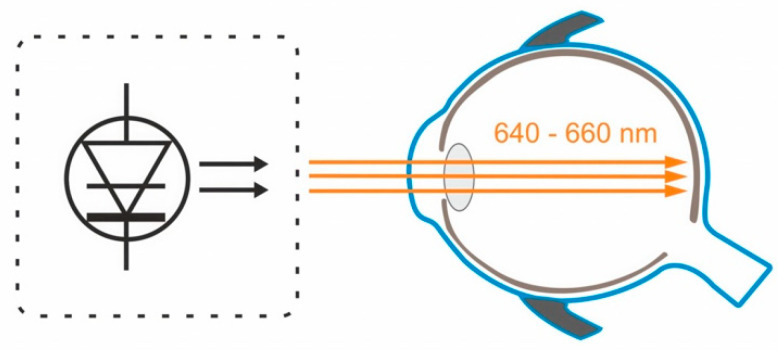
The semiconductor laser diode delivers low-level red light through the pupil to the fundus.

Figure 3 depicts the mechanism of action of low-level red light therapy device. 

Table 3 summarises the primary information from studies on the efficacy and safety of repeated low-level red-light therapy. 

## 5. The Efficacy and Safety of the Combination of Orthokeratology and Low-Dose Atropine 0.01%

In 2020, Tan Q. et al. reported the results of a one-year clinical trial that assessed the potential additive effect of 0.01% atropine eye drops in combination with orthokeratology [28]. The study included Chinese children aged 6–11 years who were randomly assigned to either the combined atropine with orthokeratology (AOK) group or the orthokeratology-only (OK) group. A total of 29 participants in the AOK group and 30 participants in the OK group completed the one-year trial. Significant differences between the groups were observed only during the first six months. Over the course of one year, the mean axial elongation in the AOK group was 0.09 mm slower than in the OK group. The authors also measured pupil size in photopic and scotopic conditions, obtaining the following results: in the AOK group, the sizes were 0.64 mm (SD: 0.48 mm) and 0.36 mm (SD: 0.34 mm), respectively, while in the OK group, they were 0.10 mm (SD: 0.50 mm) and 0.02 mm (SD: 0.28 mm), respectively.

In 2023, Tan Q. et al. published the results of a two-year randomized controlled trial comparing the combination of orthokeratology with low-dose atropine to orthokeratology alone [27]. The study included 69 Chinese children aged 6–11 years who completed the two-year study (34 in the AOK group and 35 in the OK group). Intention-to-treat and per-protocol analyses were performed, and both showed slower axial elongation in the AOK group than in the OK group. The AOK group also exhibited a larger increase in photopic and mesopic pupil size and more thickening of the choroid. The authors postulate that slower axial elongation is associated with a larger increase in photopic pupil size and more choroidal thickening.

Kinoshita et al. assessed the additive effect of orthokeratology and 0.01% atropine eye drops in myopia control in 41 Japanese children. In 2018, they published the results of the aforementioned clinical trial [26]. The study design involved all participants wearing orthokeratology lenses for three months. Afterward, they were randomly assigned to either group 1, which received orthokeratology with atropine 0.01%, or group 2, which received only orthokeratology. Axial length measurements were performed every three months, and the changes in axial length over one year were evaluated. The results showed a change of 0.09 ± 0.12 mm in group 1 and 0.19 ± 0.15 mm in group 2.

Kinoshita et al. also conducted a two-year clinical trial to assess the efficacy of combined orthokeratology with 0.01% atropine in myopia control in 80 Japanese children aged 8–12 years old with spherical equivalent refraction (SER) ranging from −1.0 to −6.0 diopters [25]. The participants were randomly assigned to either the combination group, receiving orthokeratology and 0.01% atropine once per day, or the monotherapy group, for whom only orthokeratology was used. The participants visited the clinic every three months to measure various values, including axial length, corneal endothelial cell density, intraocular pressure, uncorrected distant and near visual acuity, refraction, and corneal topography. A total of 73 children completed the study, and the effectiveness in controlling axial elongation was relatively better in the combination group, particularly in the subgroup with a lower initial SER ranging from −1.0 to −3.0 diopters.

Jiang J. et al. conducted a clinical trial to assess binocular and accommodative functions in children undergoing orthokeratology treatment combined with low-dose atropine [24]. The study included 62 participants aged 8 to 12 years old with an SER ranging from −1.0 to −6.0 diopters who were divided into four groups: the combination group (orthokeratology lenses and 0.01% atropine), the orthokeratology (OK) group (orthokeratology lenses and placebo eyedrops), the atropine group (0.01% atropine and spectacles), and the control group (placebo eyedrops and spectacles). Refraction, accommodation, and vergence function assessments were conducted before the intervention and after three months. The measured values included subjective refraction, accommodative amplitude, negative and positive relative accommodation, accommodative facility, accommodative lag, horizontal phoria, horizontal fusion vergence, and AC/A ratio. The results led to the conclusion that accommodative measurements changed in the groups using orthokeratology, whereas vergence measurements remained stable after the use of 0.01% atropine.

Zhao W. et al. described the effects of orthokeratology combined with atropine on choroidal thickness in a paper published in 2021 [32]. The analysis focused on the one-month change in subfoveal choroidal thickness (SFChT) in 154 children aged 8–12 years old with an SER ranging from −1.0 to −6.0 diopters. Among the participants, 39 used 0.01% atropine and orthokeratology, 42 used 0.01% atropine and single-vision glasses, 36 used orthokeratology and placebo, and 37 used placebo and single-vision glasses (control group). SFChT decreased in the control group and increased in all other groups.

The results of a clinical trial evaluating axial elongation and higher-order aberrations in children using orthokeratology combined with low-dose atropine were published in 2020 by Vincent SJ et al. [29]. The study included children aged 6 to 11 years old with a myopia range of −1.0 to −4.0 diopters, who were randomly assigned to either the orthokeratology group or the orthokeratology combined with atropine group. The groups consisted of 28 and 25 participants, respectively. Measurements were taken at baseline and after six months, including the amplitude of accommodation, cycloplegic spherical refraction, cycloplegic cylindrical refraction, photopic and scotopic pupil diameters, higher-order aberrations, and axial length. Data analysis revealed the following findings at six months: The photopic pupil diameter in the orthokeratology combined with atropine group was 14% larger than baseline, axial elongation was smaller in the orthokeratology combined with atropine group compared to the orthokeratology group (0.01 ± 0.12 mm vs. 0.05 ± 0.08 mm), and significant changes in ocular higher-order aberrations were noted in both groups.

A double-blinded randomized placebo-controlled trial evaluating the effectiveness of combined therapy with orthokeratology and low-dose atropine was conducted on 60 Chinese myopic children aged 8–12 years old [31]. Thirty participants received orthokeratology lenses and a low-dose atropine solution, while another 30 participants received orthokeratology lenses and a placebo solution. The participants were observed for 12 months, and axial length, pupil diameter, and accommodative amplitude were measured every four months. After 12 months, the axial elongation in the combination group was 0.10 ± 0.14 mm, while in the control group, it was 0.20 ± 0.15 mm. Significant differences were noted only in the first four months. Accommodative amplitude in both groups remained stable compared to the baseline, and pupil diameter in the control group remained stable relative to the baseline.

**Figure 4:**
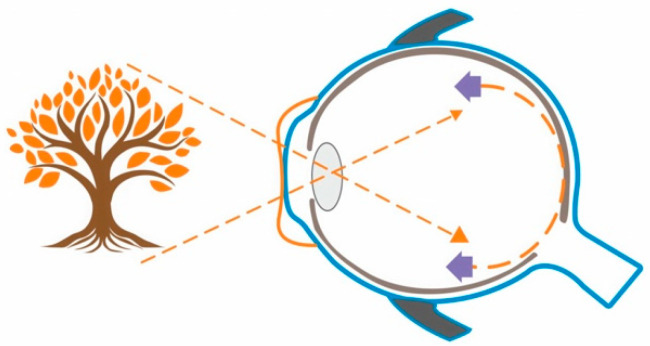
The cornea reshaped by the orthokeratology lens bends peripheral light to impose myopic defocus in the peripheral retina.

Figure 4 depicts the mechanism of action of orthokeratology lens. 

Table 4 summarises the primary information from studies on the efficacy and safety of orthokeratology combined with low-dose atropine. 

## 6. Discussion

In this review, our focus was on the latest publications regarding myopia control. It is interesting to note that the oldest publication on the topic of “myopia control” available in the PubMed database dates back to 1933 and was published in The British Medical Journal by Sorsby [34]. In this paper, various approaches to controlling myopia in schoolchildren through hygienic means were discussed. Over the years, numerous concepts and methods have emerged in an attempt to prevent the progression of myopia. While these methods described in the paper may differ in several aspects, they all share a common goal of influencing the posterior segment of the eye.

The quality of the trials described in this review is notable as they all measure myopia progression not only in terms of refraction but also by assessing the axial length of the eye. However, comparing the results accurately is challenging due to variations in materials and methods employed across the studies. While the participants in these trials are generally primary school children, the age range of participants differs among the studies. It would be valuable to assess the efficacy of the described methods in more specific age groups.

All of the studies assess cycloplegic refraction, but different protocols and drops are used. For instance, Lam C.S.Y and colleagues used two drops of cyclopentolate 1% following one drop of Alcaine 0.5% and measured refraction after 30 min. They also verified the cycloplegic effect by measuring the amplitude of accommodation with the RAF rule [10]. In a multicenter RCT on RLRL, Jiang Y and colleagues used three drops of 1% cyclopentolate at 0, 5, and 20 min, also following 0.5% Alcaine. The cycloplegic effect was confirmed by checking the pupil diameter and absence of light reflex [21]. In their study “The Efficacy of Defocus Incorporated Multiple Segments Lenses in Slowing Myopia Progression: Results from Diverse Clinical Circumstances”, Liu J. and colleagues used three drops of 0.5% tropicamide with 0.5% phenylephrine hydrochloride without verifying the cycloplegic effect through other measurements [14]. The type of cycloplegic agent used may influence the extent of cycloplegia. The measurements of choroidal thickness with optical coherence tomography can be taken with or without cycloplegia. When describing the methods used, Xiong R. indicated that cycloplegia was employed to maximize image quality and to avoid the influence of accommodation on choroidal thickness [23]. However, not all authors clearly state whether the measurements were taken under cycloplegic conditions or not.

Another limitation of many studies on preventing the progression of myopia is the lack of a double-blinding process. Implementing the double-blinding process can be difficult and expensive for certain methods. For example, the only trial on RLRL therapy performed by Dong J. et al. was double-blinded due to the use of a sham device with reduced power [19].

From a clinical perspective, it is important to consider not only effectiveness but also safety. Overall, all the described methods are considered safe, but they differ in invasiveness and time requirements. In the authors’ opinion, DIMSsl is the least invasive method, since most myopic patients are accustomed to wearing spectacles. Repeated low-level red-light therapy can be time-consuming. Orthokeratology users do not need to wear spectacles during the day, but they are required to wear contact lenses overnight. For many patients, managing contact lenses can be more burdensome than wearing spectacles during the day. Additionally, there is a risk of corneal complications associated with orthokeratology. Clearly, patients’ preferences and capabilities vary, and the optimal choice of therapy will depend on the individual needs of each patient.

## 7. Conclusions

Currently, numerous options for slowing myopia progression are emerging, but further evidence is needed to support their efficacy. Many trials have relatively short observation periods, limiting our understanding of the long-term effects. Additionally, several trials are not double-blinded, particularly in the case of repeated low-level red-light therapy. It is crucial to conduct large-scale trials involving participants from different ethnic backgrounds to ensure the generalizability of the findings. Furthermore, assessing the long-term effects of different interventions, both in monotherapy and in combination, is necessary.

Moreover, gaining a better understanding of the mechanisms of action underlying each method will provide a foundation for developing rational combined therapies. By unraveling the specific ways in which these interventions affect myopia progression, we can optimize treatment approaches and potentially enhance their effectiveness.

## Figures and Tables

**Table 1:** Search strategy.

Date of Search:	May–June 2023
Database searched:	PubMed
Target items:	Journal papers
Years covered by search:	2011–2023
Language:	English
Search terms used:	DIMS AND myopia control, Orthokeratology AND atropine AND myopia control, low-level red-light therapy AND myopia control.

**Table 2:** Studies on the efficacity and safety of defocus-incorporated multiple-segment spectacle Lenses.

Article:	Design:	Evaluated on:	Results:
Lam et al. 2020 [12]	Prospective, randomized, double-masked. Cycloplegic refraction and axial length measured at baseline and each 6-month interval over 2 years.	183 myopic children (Range: −1.0–5.0 D) Aged: 8–13 years old	DIMSsl group: myopic progression—0.41 ± 0.06 D (52% slower than SVsl) Axial length elongation—0.21 ± 0.02 mm (62% less than SVsl) SVsl group: myopic progression—0.85 ± 0.08 D Axial length elongation: 0.55 ± 0.02 mm
Zhang et al. 2020 [16]	Prospective, randomized, double-masked. Cycloplegic central refraction, peripheral refraction (at 6 retinal points (10°, 20°, and 30° nasally and temporally), and axial length measured at baseline and each 6-month interval over 2 years.	183 myopic children (Range: −1.0–5.0 D) Aged: 8–13 years old	DIMSsl group: constant and symmetrical RPR profile. SVsl group: asymmetrical peripheral myopic shifts between the nasal and temporal retina.
Lam et al. 2020 [11]	Prospective, randomized, double-masked. Distance and near best-corrected visual acuity measured monocularly; distance and near phoria; Monocular and binocular amplitude of accommodation; Lag of accommodation; Stereopsis—measured at baseline and each six-month interval over 2 years.	160 myopic children (Range: −1.0–5.0 D) Aged: 8–13 years old	After 2 years: - Distance and near phoria: no significant differences from the baseline - Slight improvement in high contrast visual acuity(DIMSsl −0.09 ± 0.07 logMAR, SVsl −0.07 ± 0.06 logMAR), - Reduction in accommodative lag (binocular AA: DIMSsl—1.90 D, SVsl—2.06 D; monocular AA: DIMSsl—1.68 D, SVsl—1.56 D), - Slight stereo-acuity improvement (DIMSsl: 5.9 s of arc, SVsl: 7.4 s of arc).
Lam et al. 2022 [10]	Prospective, not randomized. 1-year follow-up, spherical equivalent refraction, and axial length measured every 6 months. Historical control group from clinical records of the optometry clinic.	128 children: DIMS continuation *n* = 65 children, SVsl switched to DIMS *n* = 55	Myopic progression (spherical equivalent refraction) over 3 years: DIMSsl—−0.52± 0.69 D SVsl to DIMSsl—0.92 ± 0.81 D Axial length elongation over 3 years: DIMSsl—0.31 ± 0.26 mm SVsl to DIMSsl—0.57 ± 0.33 mm
Zhang et al. 2023 [15]	Prospective, not randomized. 1-year follow-up, Cycloplegic central refraction, peripheral refraction (at 6 retinal points (10°, 20°, and 30° nasally and temporally), and axial length measured at baseline and every 6-month interval for 3 years.	128 children: DIMS continuation *n* = 65 SVsl switched to DIMS after 2 years *n* = 55	DIMS group: constant and symmetrical PRP profile SVsl in the first 2 years: significant increases in hyperopic RPR at 20° nasal. After switching to DIMSsl in the third year significant reductions in hyperopic RPR at 20 N (mean difference: −1.14 ± 1.93 D, *p* < 0.0001) and 30 N (mean difference: −1.07 ± 1.17 D, *p* < 0.0001)
Lam et al. 2023 [13]	Prospective 6-year follow up. Measured values: Axial length, Cycloplegic refraction.	90 myopic children completed the study. Mean age at the enrollment was about 10 years old. Group 1: DIMSsl 6 years *n* = 36 Group 2: DIMSsl 3.5 years, SVsl 2.5 years *n* = 14 Group 3: SVsl 2 years, DIMSsl 4 years *n* = 22 Group 4: SVsl 2 years, DIMSsl 1.5 years, SVsl 2.5 years *n* = 18	SER at baseline/at 6 years for each group: Gr. 1 −3.04 ± 0.89/−3.69 ± 1.42 D Gr. 2 −2.98 ± 1.13/−4.28 ± 1.15 D Gr. 3 −2.68 ± 0.88/−3.92 ± 1.18 D Gr. 4 −2.65 ± 1.18/−3.87 ± 1.53 D AXL at baseline/at 6 years for each group: Gr. 1: 24.68 ± 0.76/25.28 ± 0.81 mm Gr. 2: 25.00 ± 0.80/25.71 ± 0.69 mm Gr. 3: 24.62 ± 0.79/25.43 ± 1.01 mm Gr. 4: 24.42 ± 0.86/25.14 ± 0.87 mm
Liu et al. 2023 [14]	Retrospective. Propensity score matching strategy.	Myopic patients 6–16 years old 3639 patients wearing DIMSsl 6838 patients wearing SVsl After PSM: 2240 pairs with one-year follow-up 735 pairs with two-year follow-up	Myopia progression in the first year: DIMSsl, –0.50 ± 0.43 D; SVsl, –0.77 ± 0.58 D; *p* < 0.001 Myopia progression in the second year: DIMS, –0.88 ± 0.62 D; SV, –1.23 ± 0.76 D; *p <* 0.001

**Table 3:** Studies on the efficacity and safety of repeated low-level red-light therapy.

Article:	Design:	Evaluated on:	Results:
Jiang et al. 2022 [21]	Multicenter, randomized, parallel-group, single-blind clinical trial Cycloplegic refraction and axial length measured at baseline and 1-, 3-, 6-, and 12-month follow-up visits.	264 children 8–13 years old with myopia from −1.0 to −5.0 D (cycloplegic SER). 117 RLRL group 129 SVsl group	Adjusted 12-month axial elongation: RLRL: 0.13 mm (95% CI, 0.09–0.17 mm) SVsl: 0.38 mm (95% CI 0.34–0.42 mm) Adjusted 12-month SER: RLRL: −0.20 D (95% CI, −0.29 to −0.11 D) SVsl: −0.79 D (95% CI, −0.88 to −0.69 D)
Dong et al. 2023 [19]	Prospective, randomized, double-blind, controlled clinical trial, Cycloplegic refraction and axial length measured at baseline and at six months.	112 Chinese myopic children 7–12 years old. RLRL group *n* = 56 Sham device control group *n* = 55	Mean SER change over 6 months: RLRL: −0.06 ± 0.03 D Sham device: −0.11 ± 0.33 D Mean AXL changes over 6 months: RLRL: −0.02 ± 0.11 D Sham device: −0.13 ± 0.10 D No treatment-related adverse events were reported
Xiong et al. 2022 [22]	Prospective, post-trial follow-up study/real-world study (RWS). Cycloplegic refraction and axial length measured at 24 months from the beginning of RCT.	114 children who completed a real-world study (after completing 1-year RCT the participants were invited to voluntarily participate an RWS). SVS-SVS group *n* = 41 SVS-RLRL group *n* = 10 RLRL-SVS group *n* = 52 RLRL-RLRL group *n* = 11	Over a 2-year period mean AXL change: SVS-SVS: −0.28 ± 0.14 mm SVS-RLRL: −0.05 ± 0.24 mm RLRL-SVS: −0.42 ± 0.20 mm RLRL-RLRL: −0.12 ± 0.16 mm Over a 2-year period mean SER change: SVS-SVS: −0.54 ± 0.39 D SVS-RLRL: −0.09 ± 0.55 D RLRL-SVS: −0.91 ± 0.48 D RLRL-RLRL: −0.20 ± 0.56 D A modest rebound effect was noted after treatment cessation.
Xiong et al. [23]	Secondary analysis of data from multicenter RCT. Values measured at 1, 3, 6, and 12 months: Changes in macular choroidal thickness (mCT) assessed by SS-OCT, Visual acuity, Axial Length, SER, and treatment compliance. Additionally: their associations with myopia control.	120 children: RLRL group *n* = 60 SVS *n* = 60	Changes in the mCT from baseline for the RLRL group: 1 month: 14,755 μm 3 months: 5286 μm 6 months: 1543 μm 12 months: 9089 μm SVS group: 1 month: 1111 μm 3 months: 8212 μm 6 months: 10,190 μm 12 months: 10,407 μm Models including only mCT changes at 3 months had acceptable predictive discrimination of good myopia control over 12 months.
Chen et al. 2022 [18]	Prospective, single-masked, single-center randomized controlled trial. Primary outcome: change in AXL Secondary outcome: change in SER followed at 1, 3, 6, and 12 months.	62 children 7 to 15 years old. Repeated Low-Level Red light (RLRL) group *n* = 31 Low-dose Atropine (LDA) group *n* = 31	Mean one-year change in AXL: RLRL: 0.08 mm (95% CI, 0.03–0.14 mm) LDA: 0.33 mm (95% CI 0.27–0.38 mm) Mean 1-year change in SER: RLRL: −0.03 D (95% CI, −0.01 to −0.08 D) LDA: −0.57 D (95% CI, −0.40 to −0.73 D)
Chen et al. 2022 [17]	Prospective, randomized, controlled clinical trial. Phase 1—treatment phase (intervention: two sessions per day lasting 3 min)–12 month Phase 2—washout phase—LRL cessation. Ophthalmic examinations at 3, 6, 9, 12, and 15 months. The outcomes: Axial length (AL), spherical equivalent refraction (SER), subfoveal choroidal thickness (SFCT), and accommodative function.	102 children 6–13 years old. Low-intensity red-light (LRL) group n = 51 Single-focus spectacles (SFS) group n =51 At 12 months completed: 46 LRL and 40 SFS	AXL elongation at 12 months: LRL: 0.01 mm (95% CI 0.05–0.07 mm) SFS: 0.39 mm (95% CI 0.33–0.45 mm) SER progression at 12 months: LRL: 0.05 D (95% CI 0.08–0.19 D) SFS: 0.64 D (95%CI 0.78–0.51 D) Changes in SFCT in the LRL group: thickening in the first 3 months, relative stability in the following months. SFS: progressive thinning of the SFCT Accommodative function assessed with: AA amplitude of accommodation AR accommodative response AF accommodative facility PRA positive relative accommodation NRA negative relative accommodation Main outcome: AR and PRA in the LRL group were more negative than in the SFS group.
He et al. 2023 [20]	Prospective, randomized clinical trial, parallel groups in 10 primary schools in Shanghai. Intervention group: RLRL twice a day 5 days per week each session lasting 3 min. Primary outcome: 12-month incidence rate of myopia (SER smaller or equal—0.5 D). Secondary outcomes: SER changes, Axial length, vision function, and optical tomography scan results over 12 months.	139 children with premyopia, primary school Grade 1–4. SER −0.5–0.5 diopter, at least one parent with SER smaller or equal −3.0 D.	12-month incidence of myopia in the RLRL group: 40.8% (49 of 120) in the control group: 61.3% (68 of 111). The RLRL intervention significantly reduced SER and AXL (myopic shifts). No visual acuity or structural damage was observed on OCT in the intervention group.

**Table 4:** Studies on the efficacity and safety of orthokeratology combined with low-dose atropine.

Article:	Design:	Evaluated on:	Results:
Tan et al. 2020 [28]	Prospective, randomized, single-masked clinical trial. Intervention: instillation of 0.01% atropine eye-drop once a day in each eye and nightly wear of 4-zone ortho-k lenses or nightly wear of 4-zone ortho-k lenses alone. After baseline 3 monthly visits for atropine prescription and ocular health monitoring. Cycloplegic examinations took place every six months. Measured parameters: refractive error, visual acuity, pupil size, amplitude of accommodation, intraocular pressure, corneal topography, axial length.	Chinese children aged 6–11 years old 29 finished 1 year trial in atropine and orthokeratology group (AOK) 30 finished 1 year trial in orthokeratology only group (OK)	Overall axial elongation in the AOK group: 0.07 (SD 0.16) mm In OK group: 0.16 (SD 0.15) mm A significant difference between groups was observed only during the first 6 months Mesopic and photopic pupil size in the AOK group: 0.64 (SD: 0.48) mm; 0.36 (SD: 0.34) mm In OK group: 0.10 (SD: 0.50) mm and 0.02 (SD: 0.28) mm
Tan et al. 2023 [27]	Prospective, randomized, single-masked clinical trial. Intervention: instillation of 0.01% atropine eye-drop once a day in each eye and nightly wear of 4-zone ortho-k lenses or nightly wear of 4-zone ortho-k lenses alone. Data collection visits took place one month after commencement and every six months later. Measurements included: refractive error, visual acuity, pupil size, and choroidal thickness (before cycloplegia).	Chinese children aged 6–11 years old 34 finished 2-year trial in the atropine and orthokeratology (AOK) group 35 finished a 2-year trial in the orthokeratology-only group (OK).	Overall axial elongation in the AOK group: 0.17 (SD 0.03) mm In OK group: 0.34 (SD 0.03) mm. Mesopic and photopic pupil size in the AOK group: 0.70 (SD: 0.09) mm; 0.78 (SD: 0.07) mm In OK group: 0.31 (SD: 0.09) mm and 0.23 (SD: 0.07) mm. Thickening of the choroid: AOK group—22.6 (SD: 3.5) μm OK group—−9.0 (SD: 3.5) μm. Adverse events: higher incidence of photophobia in the AOK group.
Kinoshita et al. 2018 [26]	Prospective, randomized clinical trial. A total of participants wore OK lenses for 3 months. Afterward, they were randomly assigned to: Group 1 receiving OK and atropine 0.01% Group 2 receiving only OK Every 3 months measurements of the AXL.	41 Japanese children 8–12 years old. SER from −1.0 to −6.0 diopters.	Axial length over 1 year: Group 1: 0.09 ± 0.12 mm Group 2: 0.19 ± 0.15 mm
Kinoshita et al. 2020 [25]	Prospective, interventional, parallel-group randomized clinical trial. Participants were randomly assigned to: Combination group (orthokeratology and 0.01% atropine) Monotherapy group (orthokeratology). Measured values: axial length, corneal endothelial cell density, intraocular pressure, uncorrected distant and near visual acuity, refraction, and corneal topography.	80 Japanese children 8–12 years old. SER from −1.0 to −6.0 diopters. 73 completed a 2-year study.	Over 2 years axial length increase: Combination gr. −0.29 ± 0.20 mm Monotherapy gr. −0.40 ± 0.23 mm AXL increase in the subgroup with initial SER from −1.0 to −3.0: Combination gr. −0.30 ± 0.22 Monotherapy gr. −0.48 ± 0.22. With Initial SER from −3.01 to −6.0: Combination gr. −0.27 ± 0.15 Monotherapy gr. −0.25 ± 0.17.
Jiang et al. 2023 [24]	Prospective, randomized clinical trial. Divided into four groups: combination group (OK lenses and 0.01% atropine), OK group (OK lenses and placebo eyedrops), atropine group (0.01% atropine and spectacles), and control group (placebo eyedrops, spectacles). Measurements at baseline and after 3 months: subjective refraction, accommodative amplitude, negative and positive relative accommodation, accommodative facility, accommodative lag, horizontal phoria, horizontal fusion vergence, AC/A ratio.	62 participants aged from 8 to 12 years old with SER from −1.0 to −6.0 completed the study.	After 3 months: - Decrease in accommodative lag in the OK group - Increase in binocular accommodative facilities and positive relative accommodations increase in combination and OK groups.
Zhao et al. 2021 [32]	Prospective, randomized, controlled trial. Group 1: 0.01% atropine and orthokeratology n = 39, Gr. 2: atropine 0.01% and single-vision glasses n = 42 Gr. 3: orthokeratology and placebo n = 36 Gr. 4: placebo and single-vision glasses n = 37 Measurements at baseline and after one month of intervention included: Subfoveal choroidal thickness, ocular biometrics, autorefraction, and best-corrected visual acuity.	154 children 8–12 years old, SER from −1.0 to −6.0 diopters.	SFChT changes: Gr. 1: 14.12 ± 12.88 μm Gr. 2: 5.49 ± 9.38 μm Gr. 3: 9.43 ± 9.14 μm Gr. 4: −4.81 ± 9.93 μm
Vincent et al. 2020 [29]	Prospective, randomized clinical trial. Assignation to OK treatment (n = 28), or OK combined with 0.01% atropine (n = 25). Measurements: photopic and scotopic pupil diameters and higher order aberrations axial length at baseline and at six months.	Children age 6–11 years old. SER from −1.0 to −4.0 diopter	Photopic pupil diameter in the AOK group: 14% larger than baseline. Axial elongation in the AOK group vs. in the OK group: 0.01 ± 0.12 mm vs. 0.05 ± 0.08 mm. In the AOK group AXL correlated with an increase in photopic pupil diameter and with some HOA metrics. The correlations mentioned above were not observed in the OK group.
Yu et al. 2022 [31]	Prospective, randomized, double-blind, clinical trial. 30 participants: orthokeratology lenses and 0.01% atropine. 30 participants: orthokeratology lenses and placebo eyedrops. Primary outcome: change in axial length (AXL). Secondary outcome: change in pupil diameter (PD) and accommodative amplitude (AMP). Measurements at 4-month intervals.	60 Chinese myopic (SER from −1.0 to −4.0 diopters) children age 8–12 years old.	After 12 months: AXL in combination group: 0.10 ± 0.14 mm In the control group: 0.20 ± 0.15 mm—significant differences only in the first four months! AMP in both groups was stable in comparison to the baseline. PD in the control group remained stable to baseline.
